# Algicidal Bacteria: A Review of Current Knowledge and Applications to Control Harmful Algal Blooms

**DOI:** 10.3389/fmicb.2022.871177

**Published:** 2022-04-07

**Authors:** Kathryn J. Coyne, Yanfei Wang, Gretchen Johnson

**Affiliations:** College of Earth, Ocean, and Environment, University of Delaware, Lewes, DE, United States

**Keywords:** algicidal, phytoplankton, bacteria–algae interactions, biological control, biofuels, harmful algal blooms

## Abstract

Interactions between bacteria and phytoplankton in aqueous ecosystems are both complex and dynamic, with associations that range from mutualism to parasitism. This review focuses on algicidal interactions, in which bacteria are capable of controlling algal growth through physical association or the production of algicidal compounds. While there is some evidence for bacterial control of algal growth in the field, our understanding of these interactions is largely based on laboratory culture experiments. Here, the range of these algicidal interactions is discussed, including specificity of bacterial control, mechanisms for activity, and insights into the chemical and biochemical analysis of these interactions. The development of algicidal bacteria or compounds derived from bacteria for control of harmful algal blooms is reviewed with a focus on environmentally friendly or sustainable methods of application. Potential avenues for future research and further development and application of bacterial algicides for the control of algal blooms are presented.

## Introduction

Phytoplankton, or single-celled microalgae, play key roles in biogeochemical cycles. They transform energy from sunlight into biomass, forming the basis of the food web in aquatic environments while providing a substantial sink for both inorganic nutrients and CO_2_. It is estimated that phytoplankton are responsible for about half of global primary production through photosynthetic activity (Falkowski, 1994; Field et al., 1998; Behrenfeld et al., 2006) and 70% of global nitrogen assimilation (Raven et al., 1993). Phytoplankton are also phylogenetically diverse, comprising both prokaryotic (cyanobacteria) and eukaryotic taxa. Based on the rate of species descriptions, it is estimated that there are approximately 70,000 species of algae from 12 phyla (De Clerck et al., 2013). Eukaryotic algae emerged through an endosymbiotic event in which a heterotrophic eukaryote engulfed a cyanobacterium, retaining its plastid (reviewed by Ponce-Toledo et al., 2019). Secondary and tertiary endosymbiotic events, followed by subsequent changes in plastid function or complete loss of plastids, gave rise to a continuum of trophic positions among algae; some species are either entirely autotrophic or entirely heterotrophic, while others occupy an intermediate trophic position that augments autotrophy with heterotrophy. Some species have also acquired the ability to steal temporary plastids from their prey through a process known as kleptochloroplasty. Although the term phytoplankton technically refers to those species that are free-floating, some photosynthetic algae also form endosymbiotic associations, for example with fungi or corals, where they provide their host with oxygen and photosynthate in exchange for protection and access to other nutrients or waste products.

While most algal species provide a benefit to the environment and other members of aquatic ecosystems, some species of algae are considered harmful. They proliferate to form dense blooms, referred to as harmful algal blooms (HABs), that negatively impact the health of the environment and aquatic species, as well as livestock and humans. Spatial and temporal increases in HABs, or “red tides,” over the past several decades have been linked to stresses associated with human activities (e.g., Vila et al., 2021), including increased eutrophication and changes in climate that promote blooms of some HAB species (Berdalet et al., 2016) (but see Hallegraeff et al., 2021). Bloom-forming species employ two strategies: production of allelochemicals that deter competitors or grazers, and/or an ability to outcompete other species for essential nutrients. The fish-killing mixotrophic dinoflagellate *Karlodinium veneficum*, for example, produces potent allelochemicals known as karlotoxins that deter grazers and immobilize its prey (Adolf et al., 2007; reviewed by Place et al., 2012; Mazzoleni et al., 2015). Algal toxins can become concentrated in higher trophic levels, leading to contamination of fish and shellfish. For instance, species within the *Alexandrium* genus produce paralytic shellfish toxins (PSTs) that can accumulate in the tissues of filter feeders such as bivalve mollusks, posing a risk to public health (e.g., Anderson et al., 2012; Bazzoni et al., 2020). HAB species are often adept at outcompeting algal cohorts, and may proliferate under both eutrophic and nutrient-poor conditions to dominate the algal community. Some species of freshwater cyanobacteria, such as the filamentous *Dolichospermum* (*Anabaena*) spp., are diazotrophs, capable of fixing nitrogen in nitrogen-depleted waters (Chia et al., 2018), while non-diazotrophs such as *Microcystis aeruginosa* activate stringent response (SR) to survive under low nitrogen conditions (Jin et al., 2020). In addition to their negative effects on health, HABs also carry socio-economic impacts due to closures of shellfisheries or economic losses among coastal communities that rely on tourism.

**FIGURE 1:**
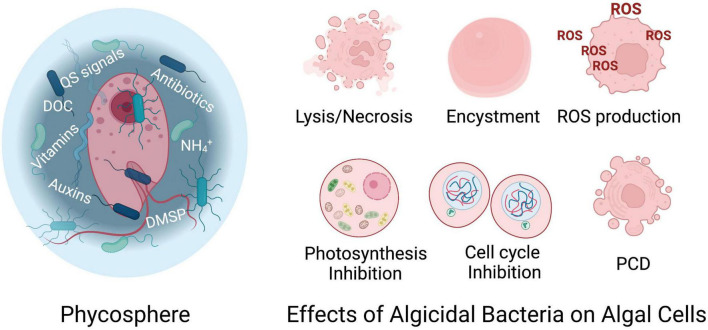
**(Left)** Bacteria-algae interactions occur in the phycosphere where chemicals are exchanged. Algicidal bacteria may exert control over algal cell growth and viability through direct physical contact or indirectly through production of algicidal compounds. **(Right)** Algal response to algicidal bacteria varies. Algicidal interactions may cause lysis, or necrosis, in which the algal cell is lysed through chemical or physical means. Algal cells may also form temporary cysts as a defense mechanism. Chemical compounds produced by algicidal bacteria may induce production of reactive oxygen species (ROS), impact photobiology, inhibit cell cycle progression and/or induce programmed cell death (PCD), or apoptosis, in phytoplankton. DOC, dissolved organic carbon, QS, quorum sensing; DMSP, dimethylsulfoniopropionate. See text for details. Created with BioRender.com.

Research and management of red tides are currently focused on the development of control or mitigation strategies that target harmful species without harming the environment or other aquatic species: approaches that would be considered environmentally friendly. One such approach to prevent or mitigate HABs is through the use of biological controls, such as bacteria. Bacteria-algal interactions are thought to occur within the “phycosphere” (Figure 1; Bell and Mitchell, 1972; Seymour et al., 2017) - a layer enriched in exudates that forms a concentration gradient of dissolved organic material (DOM) surrounding the algal cell (Amin et al., 2012). Rather than casual cohabitations, these interactions are often highly evolved and strategic. Exchanges within the phycosphere can modulate processes on local to geochemical scales (reviewed by Buchan et al., 2014), including the progression of algal blooms and the cycling of biomass through the microbial loop (Azam et al., 1983). As hosts to their bacterial cohort, algae provide a rich source of dissolved organic material in the form of fixed carbon and other organic material, while the bacteria may provide essential cofactors such as cobalamin (vitamin B_12_) or phytohormones (auxins) to stimulate algal growth and maintain the algae-bacteria relationship (e.g., Paerl, 1982; Seyedsayamdost et al., 2014; Cooper and Smith, 2015; Durham et al., 2015; Johansson et al., 2019). The essential nature of these interactions is further supported by evidence that algae actively recruit favorable bacterial colonizers to their surface by the secretion of selective chemicals (e.g., Shibl et al., 2020). Algae may also accrete bacteria that express different benefits dependent on conditions, including stresses due to light, iron, and temperature (Johansson et al., 2019). In many cases, algae suffer without their acquired bacterial mutualists and perform poorly in axenic cultures (Biondi et al., 2018). However, interactions between phytoplankton and bacteria in marine and freshwater environments are both complex and dynamic. They span a range of modes with mutualistic symbiosis on one side of this strategic spectrum, where each species benefits (reviewed by Cooper and Smith, 2015), to the opposite side of the spectrum in which pathogenic or algicidal bacteria lyse algae for access to their intracellular contents. These complex inter-kingdom relationships can fluctuate with changes in their physical and chemical environment, and are often mediated by species composition and abundance (Zhang et al., 2021c).

Several excellent reviews about algicidal bacteria in general have been published recently (Table 1). The aim of this paper is to provide a review of recent research on this topic, with a focus on algicidal interactions between bacteria and both freshwater cyanobacteria and marine HAB species. In this review, we cover our current understanding of algicidal interactions, methodological considerations for identifying and characterizing algicidal bacteria, and current implementation of algicidal bacteria for control of harmful algal blooms in the environment. We conclude with recommendations for future research to advance our knowledge of algicidal interactions in order to guide the further development of application strategies.

**TABLE 1:** Selected recent reviews of algicidal bacteria.

Topic	References
Ecological impacts of algae-bacteria interactions	Ramanan et al., 2016; Meyer et al., 2017; Cirri and Pohnert, 2019
HAB mitigation	Sun et al., 2018; Imai et al., 2021; Gallardo-Rodríguez et al., 2019
Cyanobacteria bloom mitigation	Ndlela et al., 2018; Pal et al., 2020; Yang C. et al., 2020
Quorum sensing and chemical mediators	Rolland et al., 2016; Zhou et al., 2016; Meyer et al., 2017; Wichard and Beemelmanns, 2018; Dow, 2021
Biotechnology applications	Ramanan et al., 2016; Wang et al., 2016a, 2020a; Zhang et al., 2020a

## Algicidal Interactions

**FIGURE 2:**
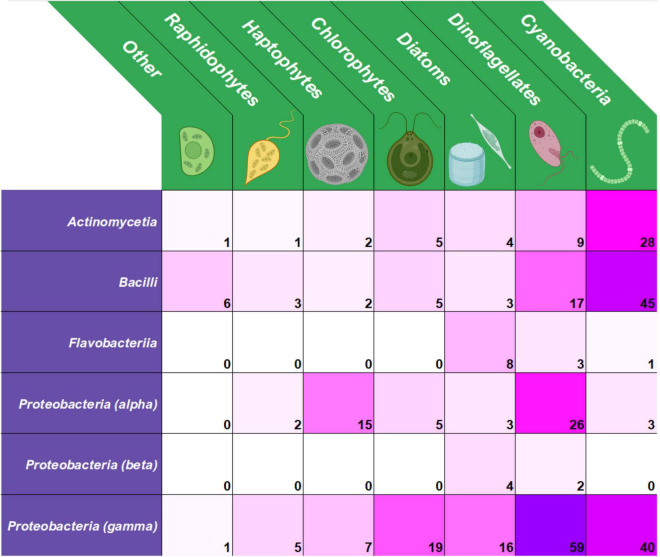
Heatmap of reported interactions between algicidal bacteria (left) and target algal groups (top). Data collected from publications in English dated 2016–2021 (Supplementary Table 1).

Antagonistic interactions in bacteria-algae relationships comprise both algicidal and algistatic interactions. The broad term “algicidal” refers to interactions that inhibit growth and/or kill algae, while the term “algistatic” more specifically connotes the inhibition of algal growth. The category of algicidal bacteria within the realm of bacteria-algae interactions is notably functional rather than taxonomic as algicidal capabilities are dispersed in bacteria without a unifying phylogenetic ancestry (Dungca-Santos et al., 2019), and groups of closely related bacteria may comprise both algicidal and non-algicidal representatives (Roth et al., 2008b; Stock W. et al., 2019). Recent publications, however, have revealed a high proportion of algicidal interactions involving bacteria from the phylum Proteobacteria, especially from the class gamma-Proteobacteria (Figure 2 and Supplementary Table 1).

**FIGURE 3:**
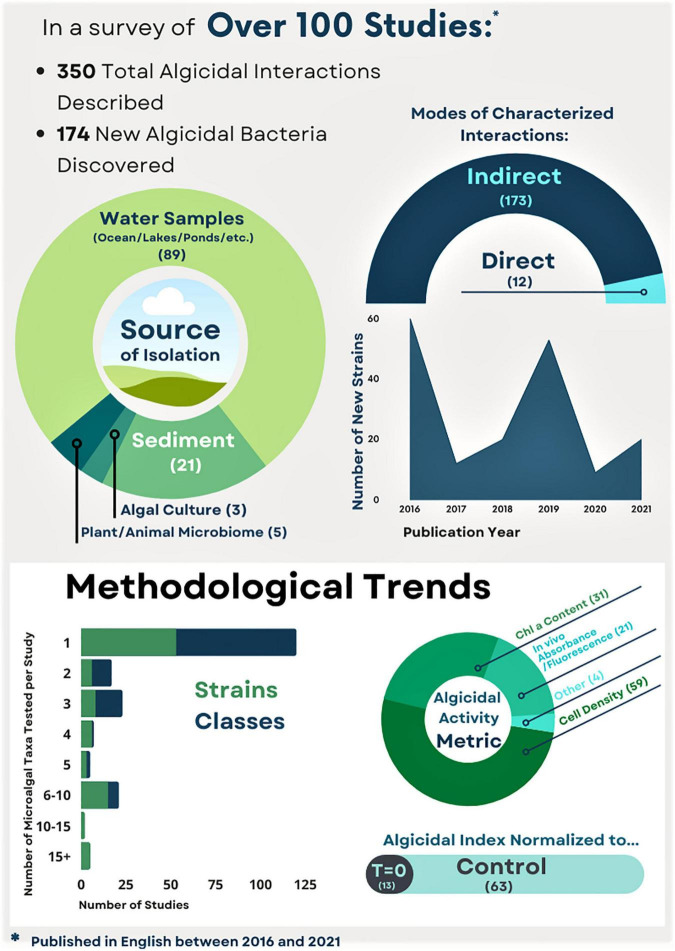
Infographic summarizing algicidal bacteria studies published in English between 2016 and 2021. Numbers of interactions and new strains, modes of attack, and methodological trends are included. For details, see doi: 10.5281/zenodo.6350760.

Algicidal interactions can also be categorized by one of two practically defined modes: indirect or direct (or both, depending on host, see Hu et al., 2019). In algicidal interactions with the indirect mode, the bacterium secretes dissolved chemicals that are algicidal. This mode of activity is demonstrated when algicidal effects on an alga can be induced by a collected exudate from which all bacterial cells have been removed by filtration. Recently, descriptions of algicidal interactions of this mode have predominated in the literature (Figure 3). The direct mode requires live bacteria cells to effectively antagonize the alga through direct contact (Zhang et al., 2019; Xue et al., 2021). For instance, Zeng et al. (2021) characterized interactions between algicidal bacterium *Streptomyces globisporus* and its target, HAB cyanobacteria *Microcystic aeruginosa*, as a direct attack, in which bacterial hyphae twined around the cyanobacteria cells. A direct contact mode may also result from the involvement of a compound that reaches effective concentrations only in the phycosphere (Wang and Seyedsayamdost, 2017). This may be due to low solubility in water, or to the localization of algicidal compounds in the bacterial cytoplasm (Jung et al., 2008). The requirement of an algal cue to induce an attack by algicidal bacteria may also result in an indirect mode of attack, but the testing of cell-free exudates collected from bacterial-algal cocultures would be needed to differentiate between such possible cases and true direct modes of algicidal interaction.

The most straightforward bacterial motive for algicidal interaction is to gain access to DOM provided upon lysis of the alga (e.g., Wang et al., 2019). The ecological rationale for algicidal interactions can be more nuanced, though. Algicidal activity may also be a way of terminating one host in the selection of a more favorable one with which it may form a longer-term association (e.g., Sison-Mangus et al., 2014; Stock W. et al., 2019). For example, Stock W. et al. (2019) found that bacteria isolated from diatoms inhibited the growth of other diatom species, as well as different strains from the same species of diatom. Indeed, when algicidal bacteria or their exudates are added to a multi-algal system, the decline of one alga is often followed by restructuring of the algal community to feature new dominant species (Tilney et al., 2014a; Fulbright et al., 2016). Host selection by a bacterium may also involve differentiating between active and senescing stages of their host, as in the case of a number of alpha-proteobacteria that are capable of killing *E. huxleyi* in response to chemical cues resulting from its structural breakdown (Wang et al., 2016b).

Taxonomic selection by bacteria is partially responsible for the observed specificity of some algicidal interactions, which may be as broad as phylum-level (Pokrzywinski et al., 2012; Wang et al., 2018) or as narrow as strain-level (Roth et al., 2008a; Barak-Gavish et al., 2018). Recent work by Shibl et al. (2020) used metabolomics combined with transcriptomics to show that the diatom *Asterionellopsis glacialis* responds to bacteria by producing secondary metabolites that promote attachment of beneficial bacteria while suppressing attachment of opportunistic bacteria. Transplanted bacteria isolated from the phycosphere of different species of *Pseudonitzschia* also showed algicidal activity against non-native hosts, while enhancing the growth of its native host (Sison-Mangus et al., 2014). This may be the result of co-adaptation of host and bacterial symbiont, and/or the ability of algicidal bacteria to switch from a mutualistic to parasitic relationship dependent on the host.

On the other hand, taxonomic specificity of algicidal interactions also results partially from defenses by the targeted alga and its mutualistic microbiome. In an example of endogenous defenses against algicidal attacks, the diatom *Chaetoceros didymus* secretes oxylipins that inhibit the growth of the algicidal bacterium *Kordia algicida* (Meyer et al., 2018). In other cases, an alga’s bacterial cohort can provide it with some relief from attacks by algicidal bacteria. *Karenia brevis* maintained without its native bacterial microbiome, for example, lost resistance to algicidal bacterium *Flavobacteriaceae* sp. S03 (Roth et al., 2008a). Understanding mechanisms that regulate algicidal specificity will improve application strategies for bacterial control of HABs (discussed below), as these interactions may be manipulated to increase specificity and reduce non-target effects on complex communities.

What has emerged from this research is a portrait of algicidal interactions that are heavily regulated and orchestrated affairs. Although the production of algicidal compounds in some species may be constitutive (Pokrzywinski et al., 2012; Whalen et al., 2018), there is abundant evidence that other bacteria exhibit activity after induction by some stimulus. This stimulus may be an algal cue that alerts bacteria to an enticing target. The dimethylsulfoniopropionate (DMSP)-metabolizing alpha-proteobacterium *Sulfitobacter* D7, for example, activated algicidal activities in response to the presence of DMSP (Barak-Gavish et al., 2018). Its algicidal activity toward different strains within the haptophyte algal species *Emiliania huxleyi* corresponded to each strain’s capacity to produce DMSP. This mode of attack was also demonstrated in Li et al. (2016), in which an algicidal bacterium exhibited chemotaxis to diatom *Thalassiosira pseudonana*. After fastening itself to the diatom frustule, the bacterium produced chitinase, which caused cell lysis and death of its diatom host. In addition, algicidal activity can be expressed over a range of intensities, becoming more potent with exposure to algal hosts (e.g., Kang et al., 2016; Weiss et al., 2019). *Alteromonas FDHY-03* upregulated beta glucosidase algicidal activity when cocultured with its target dinoflagellate *Prorocentrum donghaiense* (Shi et al., 2018), and *Alteromonas* sp. D only produced the algicidal compound Questiomycin A when exposed to the HAB raphidophyte, *Chattonella antiqua* (Umetsu et al., 2019). Algal inter-specific cues may also play a role in algicidal activity. Bigalke and Pohnert (2019) described reduced resistance to algicidal bacterium *Korida algicida* by HAB diatom *Chaetoceros didymus* when cell-free filtrates were added from a coculture between that bacterium and a susceptible diatom *Skeletonema costatum*. These examples all suggest many as-yet cryptic algae-bacterial exchanges.

There are some definitively known algicidal inducers. Seyedsayamdost et al. (2011) elegantly depicts a binary switch in the relationship between alpha-proteobacterium *Phaeobacter gallaeciensis* and coccolithophore *E. huxleyi*. *P. gallaeciensis* provides antibiotics and growth hormones to *E. huxleyi* in return for DMSP. Upon senescence, breakdown of lignin polymers in *E. huxleyi* produces p-coumaric acid, inducing *P. gallaeciensis* to divert its metabolism from the production of mutualistic products to that of Roseobacticides, potent lytic algicides. Further work demonstrated through isotope-labeling that *Phaeobacter inhibens* resourcefully incorporates p-coumaric acid from its host as an intermediate in Roseobacticide biosynthesis (Seyedsayamdost et al., 2014).

The stimulus inducing algicidal attacks may also originate from conspecific bacteria. Recent research highlights the importance of intra-taxonomic communication by bacteria, specifically quorum sensing (QS). QS is a process by which bacteria approximate their population density by reception of secreted infochemicals in order to initiate and control biochemical processes. Known types of QS signaling molecules include quinolones, acyl-homoserine lactones (AHLs), the autoinducer 2 family, and diketopiperazines, all of which contain representatives with demonstrated algicidal activity (Li et al., 2014; Harvey et al., 2016; Tan et al., 2016; Whalen et al., 2018). In addition, QS abiotic breakdown products, tetramic acids, have also been shown to have algicidal activity (Stock F. et al., 2019; Stock et al., 2020). In cases where QS molecules do not directly cause algicidal effects in algae, they may do so indirectly by controlling the onset of algicidal processes by bacteria (Guo et al., 2016; Wang et al., 2016b; Chi et al., 2017; Wu et al., 2017; Zhang et al., 2020d) or closely coupled processes, including DMSP metabolism (Johnson et al., 2016) and protease activity (Wu et al., 2017). The complex role of QS in algal-bacterial relations is reviewed in more detail in Zhou et al. (2016), Dow (2021).

Algicidal compounds that have been fully characterized reveal a high diversity in both their structural characteristics and mechanisms of action (see review by Meyer et al., 2017). Identifying the genetic components responsible for algicidal activity among bacteria provides clues to the identity of potential algicidal compounds (e.g., Hotter et al., 2021; Hu et al., 2021; Li et al., 2021d; Pal et al., 2021). This is exemplified by the genomic analysis of algicidal bacterium *Brevibacillus laterosporus* Bl-zj, which included a number of genes putatively involved in algicidal activity, including virulence factors, proteases and antibiotic resistance genes (Zhang et al., 2021a). While a full description of algicidal compounds that have been identified is beyond the scope of this review, it is worthy to note the growing realization that a number of algicidal chemicals produced by bacteria act additively or synergistically (Harvey et al., 2016; Wang and Seyedsayamdost, 2017; Zhuang et al., 2018; Ternon et al., 2019; Jeong and Son, 2021; Rose et al., 2021). These groups of chemicals may act together to overwhelm one physiological target, and/or affect multiple systems simultaneously. Wang and Seyedsayamdost (2017) elucidated a system in which the potent Roseobacticides are sometimes accompanied by Roseochelins in the algicidal interactions between *Phaeobacter inhibens* and *E. huxleyi*. While the Roseochelins have much higher IC_50_ values than the Roseobacticides, they may be auxiliary in a complex algicidal mechanism. In another recent example, Quan et al. (2021) described the effects of two algicidal compounds isolated from *Bacillus* B1 on raphidophyte alga, *Heterosigma akashiwo.* One compound, ortho-tyrosine, impacted photophysiology and membrane permeability whereas the other, urocanic acid, inhibited algal growth with no effect on membrane structure or photochemistry. Rose et al. (2021) described an algicidal cocktail composed of a group of compounds secreted by *Pseudomonas protegens* in cocultures with *Chlamydomonas reinhardtii*. These compounds, Rhizoxin S2, pyrrolnitrin, pyoluteorin, 2,4-diacetylphloroglucinol (DAPG) along with known algae-immobilizing agent orfamide A, acted differentially, with overlapping activities that produced a powerful net effect of non-motility and inhibited growth. Hotter et al. (2021) uncovered yet another toxin, the polyyne protegensin, from the antagonism between these same species that degrades carotenoid pigments, damaging *C. reinhardtii*’s eyespots and eventually causing lysis. Complementary and synergistic interactions between algicidal chemicals such as these may be optimized for the development of targeted algicidal technologies.

## Effects of Bacterial Algicides on Algae

A wide range of outcomes that occur over time scales of minutes to days have been observed for algal species after exposure to algicidal bacteria (Figure 1). These effects may lead irrevocably to cell death, as in algal cell lysis, or they may be reversible, as in formation of temporary algal cysts. Other effects may be algistatic, resulting in slowed growth of the alga in response to algicidal compounds such as cell cycle inhibitors.

Rupture of the algal cell, or lysis, is perhaps the most commonly observed effect in algicidal interactions (e.g., Zhang et al., 2017; Jeong and Son, 2021). Lysis may be an externally driven event (necrosis), induced by physical or chemical interactions resulting in the loss of membrane integrity without involving a metabolic or physiological response by the algal cell (Franklin et al., 2006). For example, mycosubtilins produced by bacteria in the genus *Bacillus* interact with the cytoplasmic membrane, resulting in increased ion permeability and lysis of both dinoflagellate and raphidophyte species (reviewed by Jeong and Son, 2021). Benzoic acid, produced by algicidal *Thalassospira* sp. also induced cell lysis in HAB dinoflagellate *Karenia mikimotoi*, possibly by passing through the cell membrane and acidifying the algal cytoplasm (Lu et al., 2016). Alternatively, lysis may occur after prolonged exposure and can be the culmination of internally driven mechanisms (reviewed by Wang et al., 2020a). For example, algicidal compounds may induce production of reactive oxygen species, leading to peroxidation of membrane lipids and cell lysis (Tan et al., 2016).

Coupling detoxification to algal cell lysis is an important consideration for control of algal blooms due to the threat of intracellular toxin release into the environment (Hagström et al., 2007; Ndlela et al., 2018). This may be a more critical factor for freshwater cyanobacteria, as the release of cyanotoxins may impact drinking water supplies. A few bacteria have been described with both algal-lytic activity against cyanobacteria, and the potential to also degrade microcystins (e.g., Li et al., 2021a; Pal et al., 2021). Genome analysis of algicidal *Rhizobium* against *M. aeruginosa*, for example, revealed the genetic capacity for algal cell wall polysaccharide degradation responsible for algal-lytic properties, along with metabolic pathways for microcystin detoxification (Pal et al., 2021). In another example, the activity of bacterium *Raoultella* sp. S1 caused flocculation of *M. aeruginosa* and degradation of photosynthetic pigments, resulting in fragmentation of algal cells and a subsequent decrease in toxin concentrations (Li et al., 2021a).

Some algae have the capacity to escape, or at least delay cell death by forming protective cysts. Approximately 200 of the ∼2,000 species of dinoflagellates, for example, are known to form cysts as part of their life cycle or in response to unfavorable environmental conditions (reviewed by Bravo and Figueroa, 2014; Jung et al., 2018). Both thin-walled “pellicle” cysts, and thick-walled “resting” cysts have been described for dinoflagellates, but the function of different morphological types of cysts is complex and not fully elucidated for all species (Bravo and Figueroa, 2014). Early work by Roth et al. (2008b) noted the presence of cyst-like cells in cultures of *K. brevis* when cultured with algicidal bacteria within the Cytophaga/Flavobacterium/Bacteroidetes (CFB) group, suggesting that algicidal bacteria may induce a transition to cyst stage in dinoflagellates. This trait may not be directly related to the activity of specific bacteria, however, but more likely represents a defense mechanism by dinoflagellates in response to algicidal activity. Proteobacterium *Pelagibaca abyssi*, for example, also induced pellicle cyst formation in the toxic dinoflagellate, *Pyrodinium bahamense* (Dungca-Santos et al., 2019). However, loss of the theca upon formation of pellicle cysts provided an opportunity for *P. abyssi* to gain entry through the thin membrane of the cyst, ultimately resulting in disaggregation of the cyst. A recent study by Prasath et al. (2021) suggests that some algicidal bacteria may also prevent the germination of cysts. Here, they described the effects of fermentation broth from *Bacillus nitratireducens* combined with coagulant polyaluminum chloride, not only to remove vegetative cells of HAB dinoflagellate *Gymnodinium catenatum*, but also prevent germination of cysts of this species.

Many algal-bacterial interactions have crystalized around metal exchanges (Amin et al., 2017; Yarimizu et al., 2018; Johansson et al., 2019), suggesting a role for metal chelators in algicidal interactions. Iron, in particular, is a crucial trace metal that limits microbial growth in the ocean (Moore et al., 2013). Most of the iron in the ocean is present as the trivalent cation, Fe(III), which is less bioavailable than the divalent Fe(II). To overcome this deficiency, marine bacteria produce a rich diversity of organic ligands that chelate trivalent iron and increase its bioavailability. Such chelates have been shown to mediate algal-bacterial iron-exchanges in some mutualisms (Amin et al., 2017; Yarimizu et al., 2018). Moreover, transcriptomic evidence suggests that chelate-mediated mutualism may play a role in facilitating some dinoflagellate blooms (Yarimizu et al., 2014). Bacterial iron chelates, however, are not universally beneficial to algae. Algal species display a range of specificity regarding the types of chelates from which they can and cannot acquire iron and heightened concentrations of even beneficial chelates can inhibit algal growth (Naito et al., 2008). Chelators may possess algicidal activity through mechanisms other than the local depletion of essential metals. The bacterial siderophore pyoverdine, for example, is capable of forming mineral nanoparticles with algicidal activity (Kumari et al., 2017). Other bacterially derived compounds with iron chelating properties have also demonstrated algicidal activity. These include tetramic acids (Kaufmann et al., 2005; Stock F. et al., 2019; Stock et al., 2020), *Pseudomonas* Quinolone Signal (Bredenbruch et al., 2006), hydroxamate siderophores (Liu et al., 2014), and Roseochelin B (Wang and Seyedsayamdost, 2017). Among these, only Roseochelin B has been suggested to serve a primarily algicidal role as opposed to quorum sensing or routine iron acquisition. Production of Roseochelin B by alpha-proteobacteria was observed in response to algal cues, but not in response to iron limitation alone (Wang and Seyedsayamdost, 2017). A notable algicidal mechanism that could be exacerbated by metal chelation is the facilitation of oxidative stress. Iron and divalent metal cations are present in the active sites of catalase and superoxide dismutase, respectively, which remediate excesses of reactive oxygen species.

The formation of reactive oxygen species (ROS) including hydrogen peroxide (H_2_O_2_), the superoxide anion (O_2_⋅^–^), singlet oxygen (^1^O_2_), and the hydroxyl radical (HO⋅) is a common occurrence for algae after exposure to bacterial algicides. ROS are produced in organisms through a number of different pathways, including electron transport reactions in photosynthesis and respiration, as well as other enzymatic reactions (reviewed by Mignolet-Spruyt et al., 2016; Dumanović et al., 2021). ROS are generated under both normal and stressed conditions and act as double-edged swords (Gupta et al., 2016; Emanuele et al., 2018). Under normal conditions, ROS initiate and participate in vital signaling transduction pathways and regulate a wide range of essential processes, including growth, development, and acclimation to stress (reviewed by Mignolet-Spruyt et al., 2016). On the other hand, overproduction of ROS leads to oxidative stress, resulting in cell damage and even cell death (reviewed by Khorobrykh et al., 2020).

This activity can have significant consequences for algicidal interactions, as an increase in intracellular ROS is among the symptoms most frequently observed in algae suffering the effects of an algicide (e.g., Li et al., 2015; Tan et al., 2016; Pokrzywinski et al., 2017b; Zhang et al., 2017, 2020b; Wang, 2021). For instance, dinoflagellates exposed to the bacterial algicide IRI-160AA exhibited increased intra- and extracellular ROS production (Pokrzywinski et al., 2017b). Subsequent transcriptomic and metabolomics analysis of HAB dinoflagellate *K. veneficium* demonstrated differential expression of key genes involved in oxidative stress and an increase in oxidative stress markers and antioxidants after exposure to IRI-160AA (Wang, 2021). Elevated ROS has also been connected to cell membrane disruption (e.g., Tan et al., 2016; Shi et al., 2018; Lyu et al., 2019; Wang et al., 2020b), chloroplast impairment (e.g., Pokrzywinski et al., 2017a), and photosynthesis inhibition (e.g., Tilney et al., 2014b; Zhang et al., 2017), among other physiological changes in algal species exposed to bacterial algicides.

The mechanism by which algicides induce the formation and buildup of ROS varies. In some cases, the production of ROS after exposure to bacterial algicides triggers cell lysis. The pigment prodigiosin, for example, is a well-documented algicidal compound produced by a number of gamma-Proteobacteria (e.g., Thomson et al., 2000; Shieh et al., 2003; Kim et al., 2006; Zhang et al., 2017). This compound has high algicidal activity against HAB species *Phaeocystis globosa*, inducing excessive ROS leading to cell lysis (Zhang et al., 2020b). Other bacterial algicides are metabolized by enzymes that produce ROS as a byproduct, such as polyamine oxidases and L-amino acid oxidase (reviewed by Moschou et al., 2008). These ROS-producing enzymes may play a large role in the algicidal activities and the ROS-mediated cell death similar to those identified in plants (reviewed by Saha et al., 2015; Gupta et al., 2016). Examples include a suite of amines produced by *Bacillus* sp. strain B1 with activity against a broad range of HAB species (Zhao et al., 2014), as well as the amine-rich exudates of *Shewanella* sp. IRI-160 that are specific to dinoflagellates (Hare et al., 2005; Ternon et al., 2019). Wang (2021) demonstrated synergistic effects of ammonium and the polyamine putrescine present in the bacterial algicide from *Shewanella* sp. IRI-160 on dinoflagellate *K. veneficum*. Subsequent transcriptomic analysis suggested that these amines together acted to disrupt polyamine homeostasis in *K. veneficum* (Wang, 2021), likely contributing to the ROS-mediated cell death in dinoflagellates exposed to this algicide (Pokrzywinski et al., 2017b).

Disruptions in photochemistry also contribute to ROS-mediated cell death. Photosynthesis requires a delicate cellular balance which can easily be disrupted, rendering algae vulnerable. It is not surprising then, that impacts on this process are commonly reported effects of algicidal bacteria on their algal target. Photosynthetic inhibition may be caused by oxidative damage or by a specific blockage in photosynthetic electron flow. The light reactions of photosynthesis, as well as photorespiration in the dark reactions, produce ROS (reviewed by Khorobrykh et al., 2020). As such, photochemical reactions may yield a positive feedback loop after initial damage by ROS, increasing ROS concentrations with degradation of photosystem function. This may be the case in some ROS-mediated algicidal mechanisms, as evidenced by a change in algicidal potency in attenuated light or darkness (Tilney et al., 2014b; Guan et al., 2015; Yu et al., 2019).

Markers for photosynthetic imbalance include both structural deformation or destruction of chloroplasts (e.g., Zhang et al., 2014; Tan et al., 2016; Pokrzywinski et al., 2017a), as well as photochemical indicators of distress (Tilney et al., 2014b; Stock F. et al., 2019; Quan et al., 2021). In some cases, algicidal chemicals from bacteria may bind to photosystem components, directly affecting photosynthetic machinery. For instance, the QS precursor, 2-heptyl-4-quinolone, produced by *Pseudomonas aeruginosa* binds directly to the cytochrome B6F complex, impeding photosynthetic electron flow in the diatom *Phaeodactylum tricornutum* (Dow et al., 2020), while tetramic acids produced by rearrangement of AHLs bind directly to the quinone binding site of PSII of this same species (Stock F. et al., 2019). Algicidal impacts on photobiology have also been illuminated by the identification of differentially expressed genes involved in photosynthesis (e.g., Zhang et al., 2020b; Wang, 2021). Transcriptome analysis of HAB species *P. globosa* after exposure to algicidal filtrates from *Bacillus* sp. LP-10, for example, revealed a downregulation of *psbA* and *rbcS* gene expression with a concomitant decrease in PSII reaction center protein D1 and an increase in ROS (Guan et al., 2015). Not all algae respond in the same fashion, even to the same algicide. For instance, species-specific responses to an algicide produced by *Shewanella* sp. IRI-160 demonstrated differential impacts on photosystem II (PSII), photosynthetic electron transport and effects of light: dark cycles on four species of dinoflagellates (Tilney et al., 2014b). Species-selective photosynthetic inhibitors such as this may hold promise as HAB mitigation tools.

Algicidal effects on cell cycle progression have also been noted. Vegetative cell division in unicellular algae is a tightly controlled process, regulated in part by nutrient availability and synchronized by entrainment of the circadian rhythm in light: dark cycles (reviewed by Huysman et al., 2014). The cell cycle in eukaryotic algae follows that in other eukaryotic species, including an S phase, in which DNA is replicated, and an M phase, where replicated copies of DNA are physically divided. These phases are separated by two gap phases, G1 and G2, which are important checkpoints in the cell cycle. Algicidal compounds associated with dysregulation of the cell cycle through changes in gene expression or protein activity can result in growth inhibition, often triggering cell death (reviewed by Bidle, 2015). Impacts on the cell cycle have been reported for several algal species after exposure to algicidal bacteria or their exudates (Pokrzywinski et al., 2017b; Stock et al., 2020; Zhang et al., 2020c; Pollara et al., 2021). Pokrzywinski et al. (2017b) used flow cytometry to examine the effects of a bacterial algicide, IRI-160AA, on dinoflagellate cell cycles. Exposure to the algicide, produced by marine bacterium *Shewanella* sp. IRI-160, induced extensive changes in cellular morphology, including chromosome decompaction (Pokrzywinski et al., 2017a). Results of this research also showed a higher proportion of cells in S phase among those treated with the algicide compared to controls, suggesting inhibition of cell cycle progression and a “pileup” of cells in the S phase (Pokrzywinski et al., 2017b). Subsequent transcriptome analysis revealed significant differences in the expression of genes involved in DNA repair, cell cycle checkpoint activation, and cell cycle regulation after exposure to the algicide compared to controls (Wang, 2021). Recent reports also implicate QS signaling compounds such as AHLs as mediators of bacteria-algae interactions with effects on cell cycle regulation (reviewed by Dow, 2021). Transcriptomic analysis of diatom *Seminavis robusta* after exposure to the AHL, oxo-C14-HSL, revealed a downregulation of genes involved in cell cycle progression, providing a causal link between AHLs and decreased cell growth by this diatom (Stock et al., 2020). Recent advances in peptide detection and quantification have also led to improved proteome analysis (e.g., Searle et al., 2018), providing additional clues to the effects of bacterial algicides on cell cycle progression. A combined transcriptomic and proteomic approach, for example, showed induction of DNA damage response accompanied by S phase arrest for coccolithophore *E. huxleyi* after exposure to bacterial QS signaling compound 2-heptyl-4-quinolone (HHQ) (Pollara et al., 2021). This effect was reversible depending on HHQ dosing concentrations, however, allowing exposed cultures to ultimately escape cell death. In another recent example, Zhang et al. (2020c) reported a tandem mass tag (TMT)-based proteomic approach to identify proteins that were differentially expressed in diatom *S. costatum* after exposure to algicidal bacterium *Halobacillus* sp. P1. Results of this study showed a significant downregulation of proteins involved in cell cycle regulation, providing molecular evidence for underlying mechanisms associated with the algicidal activity of this bacterium.

Programmed cell death (PCD) is a genetically controlled process leading to “cellular suicide” that contrasts with the externally driven process of necrosis. Originally thought to function only in metazoans, research over the past two decades has identified homologous pathways in prokaryotes, including cyanobacteria, and in eukaryotic unicellular algae (reviewed by Bidle and Falkowski, 2004; Franklin et al., 2006; Bidle, 2015). Markers for PCD include an increase in intracellular ROS, DNA degradation, caspase-like activity and externalization of phosphatidylserine, as well as distinct morphological changes associated with apoptosis, such as increased vacuolization and chromatin condensation. It should be noted that while many of these markers are shared between protists and metazoans undergoing cell death, PCD in metazoans is orchestrated and largely defined by the expression and activation of caspases, calcium-dependent proteases that cleave after aspartate residues. Homologous enzymes in phytoplankton, termed metacaspases, lack this specificity (Minina et al., 2020), along with other critical features of caspases, calling into question the involvement of metacaspases in non-necrotic cell death pathways of phytoplankton (reviewed by Berges et al., 2022). In spite of the controversy surrounding the true function of metacaspases, several studies have used aspartate-containing substrates to identify caspase-like activity accompanying cell death of phytoplankton, often associated with an increase in metacaspase expression and other markers for PCD (e.g., Wang et al., 2017; Zhao et al., 2020). There have been few investigations of PCD associated with exposure to bacterial algicides among algal species (Pokrzywinski et al., 2017b; Bramucci and Case, 2019). Pokrzywinski et al. (2017b) identified several indicators of PCD in dinoflagellates after addition of exudate from algicidal bacteria *Shewanella* sp. IRI-160, including a significant increase in caspase-3-like activity, along with DNA degradation and increased ROS. Cell death was linked to substantial impacts on chromosome morphology, suggesting that PCD in these organisms may have been triggered by the disruption of chromosome structure (Pokrzywinski et al., 2017a,b). In another example, Bramucci and Case (2019) demonstrated apoptosis-like PCD in coccolithophore *E. huxleyi* induced by algicidal bacteria *P. inhibens*. Involvement of caspase-like proteases in the death of *E. huxleyi* was demonstrated by the addition of caspase inhibitors, which successfully abolished cell death. The potential for PCD induction in HAB species by algicidal bacteria may open up additional management strategies. It is possible, for example, that degradation of cellular organelles while outer cell membranes remain intact during PCD may decrease intracellular toxin concentrations and/or prevent the release of toxins. More research is required to fully understand the role of PCD in algicide-mediated cell death for HAB species.

## Characterizing Algicidal Bacteria and Algicides

**FIGURE 4:**
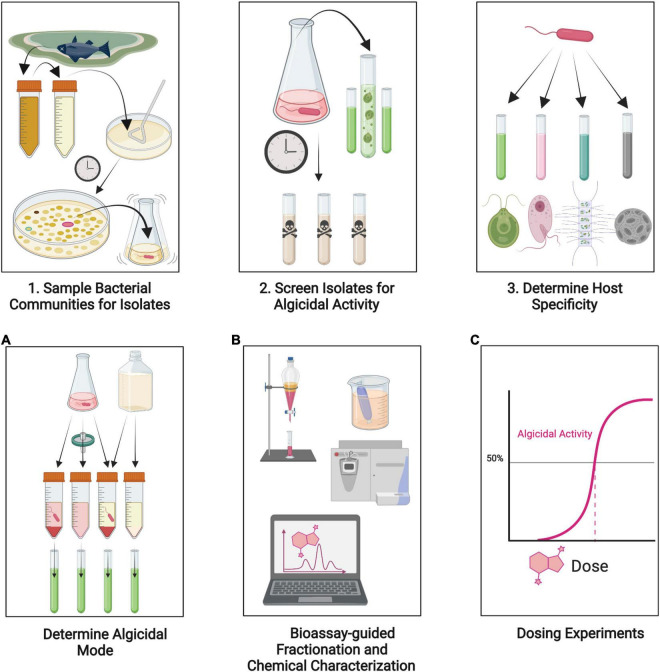
Illustration of methodological conventions for discovery and description of a new algicidal interactions. See text for details. (1) Isolate bacteria co-occurring with algae in natural blooms or in culture. Note that our knowledge of algicidal interactions is biased toward bacteria that can be cultured. (2) Conduct bioassays of isolated bacteria to screen for algicidal activity. (3) Determine host specificity and range by testing on multiple species of algae. **(A)** Determine algicidal mode by comparing activities of bacterial cell culture, cell-free culture filtrate, and washed bacterial cells. **(B)** Characterize chemicals responsible for algicidal activity. This commonly involves a bioassay-guided fractionation by solubility or polarity or by size. Further chemical characterizations may be undertaken if activity is narrowed to a fraction. The structure of compounds can be elucidated by spectroscopic methods and/or mass spectrometry. **(C)** Test the algicidal compound(s) to identify effective concentrations. Created with BioRender.com.

Increasing interest in bacterial algicides for biotic control of HABs has driven numerous efforts to identify new algicidal interactions. The work of isolation and identification of algicidal bacteria against harmful algae started several decades ago (e.g., Fukami et al., 1992; Imai et al., 1993, 1995), along with recognition of the extensive versatility of bacterial communities to produce hydrolytic ectoenzymes (Martinez et al., 1996). Since then, scores of algicidal bacteria have been described, with new isolates discovered yearly (Supplementary Table 1). Some methodological conventions have become established for preliminarily describing algicidal interactions (Figures 3, 4). Briefly, bacterial isolates are first obtained and screened for algicidal properties (e.g., Yang J. et al., 2020). Their host range, or algicidal specificity, is assessed (e.g., Pokrzywinski et al., 2012). The algicidal mode (whether or not the bacterium requires direct contact with its host) is established by comparison of the activities of bacterial culture with cell-free culture filtrate (e.g., Yang et al., 2014). With a mode of exudate, chemical characterizations and bioassay-guided fractionation of exudates may follow (e.g., Kim et al., 2015), ideally characterizing and identifying bioactive substances, the activity of which can be assessed by dose-response experiments (e.g., Yang et al., 2013).

Algicidal bacteria are often sought out intentionally by the screening of bacterial communities. These may be sampled from places where algal blooms naturally occur, such as seawater, lake water, or other aqueous environments, but bacteria from origins as unpredictable as fish intestines and Antarctic sea sponges have also displayed algicidal activity (Abdel Gawad et al., 2020; Benegas et al., 2021). Soil or estuarine sediments are also a good source of algicidal bacteria (Luo et al., 2013; Zheng et al., 2018; Zhang et al., 2021a), although the ecological functions of secreted compounds by sediment bacteria are more likely related to the high level of dissolved and particulate organic matter in this environment rather than to induce lysis in planktonic algal populations. Sampling non-axenic or poorly performing algal cultures has incidentally led to the discovery of new algicidal bacteria (Bloh et al., 2016; Fulbright et al., 2016; Hu et al., 2021). Once collected, strains are typically isolated using agar plate methods (Figure 4). It is important to note that, due to the practical constraints of this methodology, our knowledge of algicidal bacteria is overwhelmingly biased toward culturable bacteria.

A number of approaches have been used to screen for algicidal bacteria. In one approach, a lawn test is used, in which algal culture is layered onto bacterial colonies on a solid-medium plate (e.g., Kristyanto et al., 2018). The algicidal activity of an isolate is characterized by the appearance of a “zone of inhibition” around a colony, and the diameter of the zone of inhibition may be used as an indicator of the algicidal potency of each isolate (Phankhajon et al., 2016; Rose et al., 2021). However, the response by algae to algicidal bacteria may differ between liquid and agar culture (Rose et al., 2021), and coincubation bioassays in liquid culture medium are often necessary to more rigorously assess algicidal activity.

Algicidal activity is measured most often as the relative percent decrease in some algal metric. The two most common metrics for assessing algicidal activity are algal cell abundance and extracted pigment content (usually chlorophyll *a*). Other metrics including dry weight and *in vivo* pigment fluorescence are used with less frequency (Figure 3). The selection of this metric, and whether its value in the treatment group at the time of measurement (T_i_) is normalized to initial (T_0_) concentrations, or to the control group at T_i_ has important implications. In addition, comparisons to control cultures at T_i_ do not take into account the growth and mortality of treatment and control cultures, making it difficult to distinguish between algicidal and algistatic activities. Moreover, cell density-based algicidal metrics normalized to T_0_ are heavily biased toward identifying lytic phenomena. Interactions with non-lytic effects, which are of interest for toxigenic HAB species, may be overlooked if algicidal effects are identified only as a relative decrease in cell density. Harvey et al. (2016), for example, showed that algicidal compounds released by *Pseudoalteromonas piscicida* significantly inhibited the growth of *E. huxleyi*, inducing mortality without cell lysis. The most informative approach to determining algicidal activity includes pre- and post-incubation values for both treatment and control groups. Furthermore, while preliminary investigations often use just one algicidal metric, more extensive characterizations of algicidal/algistatic activity may require measurement of two or more metrics (e.g., Tilney et al., 2014b).

Establishment of algicidal specificity requires exposing multiple taxonomically diverse algae to the bacterium under scrutiny (Figure 4). The number of taxa that have been used to determine specificity varies widely (Figure 3). Still, the host specificity of many algicidal bacteria is not known; nearly half of studies evaluated in this literature review used only one strain of algae for their bioassays and further specificity testing has not yet been published. Finally, testing of taxonomic specificity may be repeated after identification and purification of algicidal compounds as pure compounds sometimes demonstrate taxonomic specificity that a raw exudate does not (Lin et al., 2015; Harvey et al., 2016).

## Control of Harmful Algal Blooms by Algicidal Bacteria in the Environment

While laboratory culture experiments demonstrate the potential for control of algal species by algicidal bacteria, these experiments are often conducted with a single species of algae paired with a single bacterial species under highly controlled and often artificial conditions (reviewed by Mayali and Azam, 2004). In addition, laboratory culture experiments commonly use cell densities that far exceed those that would be encountered in the environment, raising the question of whether encounters and interactions between algicidal bacteria and target algal species at environmentally relevant concentrations would occur at a rate necessary to affect blooms. Seong and Jeong (2013) determined that the lowest concentrations of a broad spectrum of algicidal bacteria species required to kill their intended target ranged from 3.7 × 10^3^ to 1.3 × 10^6^ cells mL^–1^. Other biotic and abiotic interactions can also play role in algicidal activity in the field. Examples include interactions between bacterial cohorts, or conditions that introduce secondary stressors, which are not typically examined in laboratory culture experiments but may be highly relevant in natural settings (discussed in more detail below).

Algicidal interactions that rely on the activity of dissolved compounds may also be less effective in the field due to dilution by the surrounding aqueous environment. However, many motile bacteria exhibit chemotaxis toward exudates from phytoplankton, increasing the rate of encounters (reviewed by Seymour et al., 2017). Interactions that require direct attachment of bacteria to the target algal cell may concentrate algicidal bacteria within the phycosphere, increasing rates of algicidal interactions (Mayali et al., 2008). Assessment of algicidal activity among isolates from a diatom bloom supported this hypothesis, with nearly five times the number of particle-associated bacteria exhibiting algicidal activity compared to free-living isolates (Park et al., 2010). Recent work also demonstrated that algicidal bacteria may concentrate within specific environments or habitats, where their abundance would increase the potential for control of algal blooms. Free-living (Sakami et al., 2017) and epiphytic bacteria with algicidal and antifouling properties are abundant on macroalgae or seagrasses (Inaba et al., 2017; Imai et al., 2021), and there is evidence that they may regulate biofouling by algae (reviewed by Tarquinio et al., 2019). Negative correlations between seagrass beds and algal abundance further suggested that algicidal bacteria associated with these beds may have some control over the growth of algae in the environment (Inaba et al., 2018; Onishi et al., 2021), and the potential for development of management strategies to control HABs (Imai et al., 2021), as discussed in more detail below.

Perhaps the most convincing evidence for control of HABs by naturally occurring algicidal bacteria in the environment is the presence and increased abundance of algicidal bacteria during late stages of blooms (e.g., Imai et al., 2001; Kim et al., 2008; Liu et al., 2008; Park et al., 2010; Zhang et al., 2012; Inaba et al., 2014; Scherer et al., 2017). Indeed, sampling the bacterial community during blooms is an effective approach to isolate algicidal bacteria from the environment (e.g., Rashidan and Bird, 2001; Park et al., 2010; Ismail and Ibrahim, 2017; Shi et al., 2018; Zhang et al., 2019; Cho et al., 2021). For instance, Shi et al. (2018) recently described an algicidal gamma-Proteobacteria in the genus *Alteromonas* isolated during a bloom of HAB dinoflagellate *P. donghaiense*. Laboratory culture experiments subsequently showed that this bacterium upregulated beta-glucosidase expression in the presence of the dinoflagellate, resulting in digestion of the alga’s cell wall. The abundance of other algicidal bacteria may not fluctuate during blooms, but their prevalence in the microbial community suggests that they play a role in bloom dynamics (Scherer et al., 2017).

Direct evidence for control of blooms by algicidal bacteria, however, is limited. Early work by Skerratt et al. (2002) investigated several species of algicidal bacteria that were isolated during a bloom of HAB dinoflagellate *G. catenatum*, showing that concentrations of algicidal substances produced by these bacterial species could be effective at controlling *G. catenatum* growth if they dominated the bacterioplankton population. As noted above, Adachi et al. (1999) showed a significant correlation between HAB dinoflagellate *Alexandrium tamarense* and cyst-promoting bacteria at bloom sites, suggesting that activity by this bacterial consortium may affect bloom dynamics, leading to the demise of *Alexandrium* blooms in the environment. Cyst-promoting activity in the field is also supported in a recent report by Dungca-Santos et al. (2019), in which bacteria isolated from algal blooms induced cyst formation in culture experiments. Several reports also provide evidence that heterotrophic bacteria capable of degrading cyanotoxins increase in abundance during blooms of cyanobacteria. For instance, microcystin-degrading bacteria increased upon bloom senescence, when intracellular toxins were released by cyanobacteria (Omidi et al., 2021).

Other work showed that bacteria exhibiting algicidal activity in laboratory culture may not respond to algal blooms in the natural environment. Jones et al. (2010), for example, evaluated the relative abundance and diversity of bacteria during blooms of *K. brevis* compared to low-abundance or non-bloom conditions. Bacteria known to be algicidal from laboratory culture experiments were identified in low abundance during bloom and non-bloom conditions, suggesting that these species do not play a significant role in regulating *K. brevis* blooms in the environment, and/or that other biotic interactions may control their abundance (Mayali and Doucette, 2002). Abiotic factors and environmental conditions have also been shown to regulate algicidal activity of bacteria. Bacteria algicidal toward the HAB dinoflagellate *Alexandrium catenella*, for example, expressed algal-lytic properties only when cultured in high-nutrient media, and displayed no adverse effects on *A. catenella* when added to cultures that were free of organic nutrients (Amaro et al., 2005).

## Application Strategies for Control of Harmful Algal Blooms

**FIGURE 5:**
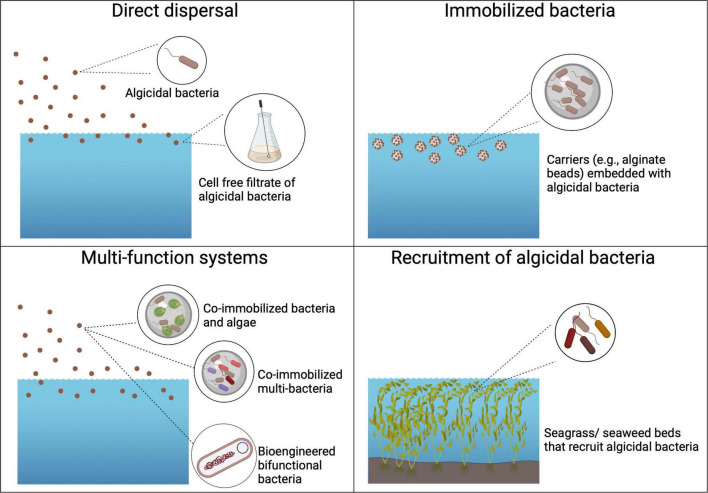
Potential application strategies of algicidal bacteria for HAB prevention and mitigation. Current and proposed strategies include direct dispersal of algicidal bacteria and/or their algicides, application of immobilized algicidal bacteria, deployment of multi-function systems, and restoring seagrass/seaweed beds to recruit algicidal bacteria. See texts for details. Created with BioRender.com.

With limited direct evidence for control of HABs by naturally occurring algicidal bacteria, there is growing interest in both research and management to develop application strategies for the use of algicidal bacteria or their products to control HABs (e.g., Imai et al., 2021; Mehrotra et al., 2021). These application strategies include direct dispersal of bacteria and/or their algicidal products, deployment of immobilized algicidal bacteria for more targeted dispersal, the use of multi-functional systems, and deployment of substrates such as seagrass beds to recruit naturally occurring algicidal bacteria (Figure 5). It is unlikely that any one approach will be appropriate for all HABs. Management strategies to control HABs will require an assessment of cost, location, feasibility, social acceptance, and target species when deciding on an appropriate control method (Hudnell, 2010; Brooks et al., 2016).

The application of algicidal bacteria and their algicides in their native environment is often cited as an “environmentally neutral” means to control HABs. So far, however, few studies evaluated these biotic agents in natural communities (Table 2). For instance, Tilney et al. (2014a) investigated the effects of the cell-free filtrate (IRI-160AA) of algicidal bacterium *Shewanella* sp. IRI-160 in laboratory microcosm experiments initiated from dinoflagellate blooms. Results of this research demonstrated the efficacy of IRI-160AA on the targeted dinoflagellate species in the collected field water, although a lower algicidal activity was observed compared to the laboratory monocultures. Moreover, while dinoflagellate abundance was inhibited by the algicide exposure, diatoms and ciliates increased in abundance, implying the algicide produced by *Shewanella* sp. IRI-160 may provide a benefit to other phytoplankton and eukaryotic organisms. This research highlighted the potential effects of microbial community complexity on the efficacy of bacterial algicides, and provided insights on the potential impacts of this algicide at a community level. Similar to Tilney et al. (2014a), the majority of research evaluating the effects of algicidal bacteria on natural communities has been conducted under controlled conditions in the laboratory (Jung et al., 2008; Tilney et al., 2014a; Kang et al., 2016; Su et al., 2016; Grasso, 2018).

**TABLE 2:** Select studies on algicidal bacteria or their filtrate using natural communities.

Bacterial species	Application method	References
*Lactobacillus paraplantarum* KCTC 5045^T^	Laboratory	Kang et al., 2016
*Acinetobacter* sp. J25	Laboratory	Su et al., 2016
*Shewanella* sp. IRI-160 (cell filtrate)	Laboratory	Tilney et al., 2014a; Grasso, 2018
*Pseudomonas fluorescens* HYK0210-SK09 (SK09)	Laboratory	Jung et al., 2008; Jung et al., 2010
*Pseudomonas fluorescens* HYK0210-SK09 (SK09)	River enclosures	Jung et al., 2010; Kang et al., 2011; Noh et al., 2017

Only a few investigations have been conducted in open water in the field. To date, all reports on field applications of algicidal bacteria involved the use of bacterium *Pseudomonas fluorescens* HYK0210-SK09 (SK09) in river enclosures (Jung et al., 2010; Kang et al., 2011; Noh et al., 2017). These reports underline the necessity of conducting field trials to confirm activity under conditions that may differ from those of laboratory culture experiments. Although SK09 exhibited strong species-specific algicidal activity against the winter-blooming harmful diatom *Stephanodiscus hantzschi* in laboratory culture experiments and indoor mesocosm studies (Jung et al., 2008, 2010), experiments conducted in less controlled field environments yielded inconsistent results (Jung et al., 2010; Kang et al., 2011; Noh et al., 2017). Temperature likely played a role in the efficacy of SK09 along with other factors, including the high concentrations of algicidal bacteria required (Seong and Jeong, 2013). Grazing pressure by other protists such as heterotrophic flagellates and ciliates (Jung et al., 2010; reviewed by Noh et al., 2017) may also play a crucial part in the success of algicidal bacteria.

Lessons learned in the transfer of controlled laboratory studies to the field highlight the major challenges and complexities surrounding the application of algicidal bacteria or their algicides to control HABs. pH, nutrient status, and the presence of beneficial bacteria, in addition to the environmental factors mentioned above (temperature and grazing pressure), may all negatively affect the ability of the algicidal bacteria to control HABs (reviewed by Ndlela et al., 2018; Yang C. et al., 2020).

Biosafety concerns may also be raised with the large-scale dispersal of bacteria, even when shown to be naturally occurring. For instance, *Vibrio*, *Shigella* and *Alcaligenes* species are naturally occurring bacteria with algicidal activity (Seong and Jeong, 2011; Xue et al., 2021), but also pose a danger to human health and marine life. *Vibrio parahaemolyticus*, for example, exhibited potent algicidal activities against harmful dinoflagellates (Seong and Jeong, 2011), but is also pathogenic to marine bivalves (Turner et al., 2016), and can cause wound infection and food poisoning in humans (reviewed by Letchumanan et al., 2014). *Vibrio brasiliensis* also exhibits algicidal activity against HAB species (Ouyang et al., 2021), but is pathogenic to shrimp (Li et al., 2021b). Any environmental benefit to the direct dispersal of these and other pathogenic bacteria is likely to be outweighed by the risk to human health, so that alternative deployment strategies should be adopted for their environmental application.

An alternative to direct dispersal of algicidal bacteria is the use of a matrix to concentrate and immobilize bacteria for deployment in areas at risk of HABs (Table 3). Bacteria are commonly found associated with surfaces in nature, which provide them with a number of benefits including protection from adverse environmental conditions and predators (e.g., Main et al., 2015), as well as increasing access to nutrients (reviewed by Żur et al., 2016). Taking advantage of this characteristic, bacteria have been immobilized by embedding or encapsulating into porous matrices to prevent dispersal (reviewed by Mehrotra et al., 2021). Bacterial immobilization technology has been utilized in a wide range of disciplines to enhance bacterial activity in a number of applications, including wastewater treatment (e.g., Hou et al., 2020, 2021), degradation of marine oil pollution (e.g., Li et al., 2021c), and probiotic delivery (e.g., Dimitrellou et al., 2019; reviewed by Mettu et al., 2021). Algicidal bacteria have also been immobilized to various matrices for HAB mitigation (Table 3), including immobilization in (polyvinyl alcohol-) sodium alginate (e.g., Su et al., 2017; Zhang et al., 2018; Wang and Coyne, 2020; Ouyang et al., 2021), cellulose sponge (e.g., Noh et al., 2017; Wang and Coyne, 2020), polyester (e.g., Wang and Coyne, 2020), polyurethane (e.g., Kang et al., 2007), agarose (e.g., Wang and Coyne, 2020), agar (e.g., Kang et al., 2007), and coconut fibers (e.g., He et al., 2021).

**TABLE 3:** Select recent studies on immobilization of algicidal bacteria.

Bacterial species	Matrices	Addition	References
*Acinetobacter* sp. J25	Sodium alginate	Fe_3_O_4_	Su et al., 2017
*Bacillus methylotrophicus* ZJU	Sodium alginate	Fe_3_O_4_ and wheat bran	Sun et al., 2016
*Alcaligenes aquatilis* F8	Sodium alginate	wheat bran	Sun et al., 2015
*Shewanella* sp. IRI-160	Sodium alginate, cellulose sponge, polyester, and agarose	/	Wang and Coyne, 2020
*Brevundimonas* sp. AA06	Polyvinyl alcohol-sodium alginate	/	Zhang et al., 2018
*Pseudomonas fluorescens* SK09 HYK0210- SK09 (SK09)	Activated carbon polyvinyl alcohol sponge	/	Noh et al., 2017
*Vibrio brasiliensis* H115 (fermentation products)	Sodium alginate	CaCO_3_, citric acid, and ethylcellulose	Ouyang et al., 2021
Mixed bacteria from eutrophic water	Coconut fiber	/	He et al., 2021

Among the carriers for algicidal bacteria immobilization, sodium alginate is the most extensively evaluated and most widely used matrix (Table 3). Alginate is a natural polymer secreted by bacteria and brown algae, and is low-cost, non-toxic, and biodegradable (reviewed by Lapointe and Barbeau, 2020). Because of these features, alginate is considered both suitable and environmentally neutral as a carrier to deliver algicides from bacteria. Immobilization of algicidal bacteria typically involves mixing cultures of bacteria with an alginate solution, and dropping the mixture into a cold CaCl_2_ solution, forming crosslinked hydrogel beads with bacteria embedded (e.g., Kang et al., 2007; Sun et al., 2015; Wang and Coyne, 2020). The formed alginate beads contain nanopores (reviewed by Lee and Mooney, 2012), which allow the diffusion of small molecules, including bacterial algicidal compounds (e.g., Sun et al., 2016; Wang and Coyne, 2020). The retention of algicidal bacteria within alginate beads may also prevent the release of high densities of bacteria into the environment as these beads can be retrieved after application, and thus avoid the biosafety concerns regarding the direct dispersal of large quantities of bacteria. For instance, Wang and Coyne (2020) evaluated alginate along with agarose, cellulose sponge, and polyester webbing as a carrier to immobilize the algicidal bacterium *Shewanella* sp. IRI-160. They demonstrated a superior packaging capacity of alginate hydrogel compared to the other carriers, where over 99% of the bacteria were retained in the alginate beads after 12 days (Wang and Coyne, 2020). The same study demonstrated *Shewanella* sp. IRI-160 embedded in alginate beads were as effective as their free-living counterparts with respect to their algicidal effects against targeted dinoflagellates (Wang and Coyne, 2020).

Alginate hydrogel also has the advantage that it can encapsulate other additives with the algicidal bacteria to allow better algicidal activity or enhance features of the beads. For instance, Sun et al. (2015) increased the ability of immobilized algicidal bacterium *Alcaligenes aquatilis* F8 to control the growth of HAB cyanobacterium *M. aeruginosa* by adding wheat bran to alginate hydrogel beads. The resultant higher algicidal activity may have resulted from an enhancement of bacterial growth within the matrix due to multiple vitamins provided by the wheat bran (Sun et al., 2015). A similar study was conducted on immobilized algicidal bacterium *Bacillus methylotrophicus* ZJU in alginate hydrogel beads against *M. aeruginosa* (Sun et al., 2016). In addition to wheat bran, Sun et al. (2016) also encapsulated Fe_3_O_4_ nanoparticles, which allowed the retrieval of the immobilized bacteria using a magnet. Moreover, recent research indicated a superior performance of alginate hydrogel when immobilizing products of algicidal bacteria instead of the bacteria itself. Ouyang et al. (2021) optimized performance of alginate hydrogel embedded with spray-dried cell-free fermentation broth from algicidal bacterium *Vibrio brasiliensis* H115, and included CaCO_3_ in the matrix as a CO_2_ gas-forming agent to add buoyancy. The encapsulation of the dried algicidal products not only had a potent algicidal activity against the targeted HAB dinoflagellate *Akashiwo sanguinea*, but could also float to control blooms at the water surface (Ouyang et al., 2021).

The choice of matrix for immobilization of algicidal bacteria must be determined empirically based on characteristics of algicidal compounds and the mode of algicidal interactions. For instance, Kang et al. (2007) immobilized the algicidal bacterium *P. fluorescens* HYK0210-SK09 into different matrices, including alginate, agar, cellulose sponge, and polyurethane, and observed much lower inhibiting effects by bacteria embedded into alginate hydrogel on the targeted diatom *S. hantzschii* compared to the free-living bacteria and those immobilized into other matrices tested. In contrast, Wang and Coyne (2020) demonstrated higher activity of algicidal bacteria *Shewanella* sp. IRI-160 immobilized in alginate beads compared to other matrices tested. Differences in results highlight the importance of evaluating efficacy for a range of matrices. Algicidal compounds secreted by bacterium *Shewanella* sp. IRI-160 were characterized as hydrophilic small molecules (Pokrzywinski et al., 2012; Ternon et al., 2019), so that immobilization to the nanoporous alginate hydrogel did not impede the release of these algicides (Wang and Coyne, 2020). Conversely, the algicidal compounds produced by *P. fluorescens* HYK0210-SK09 were localized in the cytoplasm, and required direct attachment of the bacteria for algal lysis (Jung et al., 2008). In this example, carriers such as cellulose sponge that have larger pores compared to the alginate hydrogel may enhance interaction between *P. fluorescens* HYK0210-SK09 and the targeted diatoms to permit higher algicidal activities (Kang et al., 2007).

Multi-function systems, such as co-immobilized multi-species, as well as algicidal bacteria engineered to achieve multiple functions, can also be explored as tools for control of HABs using algicidal bacteria. Co-immobilization of mutualistic bacteria with algae has been shown to promote algal growth and the production of algal by-products (reviewed by Fuentes et al., 2016). This technology has also evaluated for wastewater treatment (reviewed by Zhang et al., 2020a), where mutually symbiotic interactions enhanced the growth of both microorganisms and resulted in better nutrient absorption compared to the single-organism systems (e.g., Shen et al., 2017). To date, the use of immobilized algae alone or co-immobilized with bacteria has not been investigated for the purpose of HAB prevention and mitigation. Future studies may focus on the development of application strategies using immobilized non-harmful algae and HAB-specific algicidal bacteria as a potential dual-purpose system to prevent and control bloom events, for example by reducing nutrients and directly controlling the growth of HAB species.

Cell-lysis, reviewed above, is the most frequently described outcome for interactions between algicidal bacteria and their algal targets. Although very effective, there are concerns about the release of toxins by cell disruption; in fact, increased toxin release in the water upon algal cell lysis has been considered as counterproductive to algal mitigation efforts, and represents a major limitation for the environmental application of algicidal bacteria (reviewed by Ndlela et al., 2018). To address this issue, methods have been proposed to apply bifunctional systems that have the ability to both lyse cells and to degrade any toxins that are released (reviewed by Sun et al., 2018). Although prior research shows that dual functions may be realized by a single algicidal bacterium (e.g., Li et al., 2021a), or that some algicides reduce the toxic potential of algae even when lysed (Cai et al., 2016; Zhuang et al., 2018; Yu et al., 2019), the combined attributes of lysis and toxin degradation may be more readily achieved through immobilization of multiple bacteria, each with different functionality. This approach was proposed by Lee et al. (2018), who isolated *Bacillus* sp. T4 with algicidal activity against *M. aeruginosa* along with several other bacterial species that had the capacity to degrade microcystin toxins. The application of multiple bacteria to achieve multiple functions such as this, however, has yet to be demonstrated in the environment.

Another approach with demonstrated potential is the development of bifunctional algicidal bacteria through genetic engineering (reviewed by Sun et al., 2018). Genetic engineering involves the utilization of recombinant DNA from different species to produce hybrid organisms with the potential for additional, non-native functions (reviewed by Robert and Baylis, 2008; Rosenberg, 2017), or DNA mutation within a single species to improve enzyme activity or broaden functionality (e.g., Macdonald et al., 2016). Recent advances in genetic engineering tools make this process more precise and efficient, while also less labor-intensive (reviewed by Yan and Fong, 2017). Recent research demonstrated the feasibility of this approach for genetically engineered algicidal bacteria. For instance, Macdonald et al. (2016) described a polysaccharide lyase with exolytic properties from the gamma-Proteobacteria, *Stenotrophomonas maltophilia*, that was engineered to achieve additional substrate specificity and more efficiently disrupted algal cell walls. Further research by Eckersley and Berger (2018) demonstrated the efficacy of this engineered enzyme to lyse and inhibit the growth of *M. aeruginosa*. To date, no research has demonstrated such an engineered bacterium with dual functions. Application of these engineered microorganisms will also require extensive efforts to evaluate biosafety and their uncertain environmental impacts. Methods such as immobilization may be explored in the future to protect these bioagents and allow their use in targeted field applications.

A viable alternative to the application of laboratory-cultured algicidal bacteria for control of HABs is enhancing the recruitment and growth of algicidal species to areas that are at risk of HABs. Seagrass and seaweed beds, as mentioned above, are hotspots for bacteria that inhibit the growth of microalgae (Sakami et al., 2017). Biofilms composed of complex communities of epiphytes including algicidal bacteria are recruited to the surfaces of seagrasses and macroalgae, which in turn provide a continuous supply of these species back into the surrounding seawater for HAB prevention and mitigation (also see detailed review by Imai et al., 2021). The surface of seagrass and macroalgae not only provide habitats for bacteria, but may also exude nutrients that promote bacterial growth (reviewed by Tarquinio et al., 2019). The density of algicidal bacteria inhabited in the seagrass leaves can be over 10^7^ colony-forming units (CFU)/g of the leaf wet weight (e.g., Inaba et al., 2017). A study conducted by Inaba et al. (2020) using artificially created *Ulva pertus*a beds with floating cages demonstrated a much higher density of bacteria with algicidal activity against raphidophytes and dinoflagellates on the macroalgae beds compared to the surrounding seawater. A similar level of algicidal bacteria was found on the artificial macroalgae beds compared to those that are natural in the environment (Inaba et al., 2020). These results indicated the potential of using artificially introduced macroalgae beds to recruit algicidal bacteria as a boost to HAB mitigation in areas at risk for toxic blooms, such as shellfish harvesting sites. In addition to HAB mitigation, seagrass and seaweeds provide essential habitat for wildlife and reduce nutrients (reviewed by Morris et al., 2018) while also mitigating coastal hazards by reducing wave heights and preventing floods (reviewed by Morris et al., 2018; Valdez et al., 2020). Overall, restoration of seagrass and macroalgae may provide an ecologically friendly means to mitigate HABs. A drawback to this approach compared to direct application of specific algicidal bacteria may be the time it takes for algicidal bacteria to become established. However, as mentioned by Imai et al. (2021), little effort is involved in maintaining seagrass and macroalgae beds once they are restored, so that this method may also represent a cost-efficient approach for HAB control efforts.

## Future Avenues for Research and Development of Application Strategies

A limited number of harmful algal species have been used in studies to evaluate algicidal interactions with bacteria, making it difficult to compare across studies for specificity and potency of algicidal interactions. For this reason, a small number of representative algal isolates from major taxa could be established in the future to make testing more uniform and specificity of certain algicides more comparable between studies. This practice may also potentially uncover new applications for already isolated and well-studied bacteria. Additionally, the release of toxins from toxic algal species after exposure to bacterial algicides may be a concern, delaying development of field application. Future research should be directed to isolate algicidal bacteria that degrade algal toxins or limit their release into the environment.

The application of algicidal bacteria in biotechnology provides a rich source of information that could be directed toward the development of control strategies for HABs. Research has long demonstrated the value of microalgae and microalgae-based products in a variety of fields, including pharmaceuticals, cosmetics, biofuels, and food industries (Saini et al., 2020; reviewed by Barati et al., 2021; see reviews: Khavari et al., 2021; Orejuela-Escobar et al., 2021). Pretreatment of algae to disrupt cell walls is an essential step for the extraction of valuable compounds, and substantial effort has been directed toward using biological means, including the application of algicidal bacteria, to disrupt cell walls (Demuez et al., 2015; Wang et al., 2020a). Recent examples of this approach for algal lipid production include the use of *P. pseudoalcaligenes* AD6 and *Aeromonas hydrophila* AD9, both isolated from shallow wetland sediments (Lenneman et al., 2014), *Paracoccus* sp. strain Y42 (Gui et al., 2021), *Labrenzia* sp. for lysis of diatom *P. tricornutum* (Chen et al., 2015), and *Sagittula stellata*, which was isolated from an area with persistent algal blooms (Wang et al., 2021). These bacteria were highly effective in disrupting the algal cell walls, allowing the efficient extraction of the lipid products. While objectives differ, additional data sharing between members of the HAB research community and the growing microalgal biotech industry has the potential to spur development of algicidal bacteria and improve application strategies to control algal blooms.

To date, only limited studies evaluated the effects of bacterial algicides on non-target species, especially those at higher trophic levels (e.g., Chen et al., 2021; Simons et al., 2021). Simons et al. (2021) investigated the effects of the algicides (IRI-160AA) produced by the bacterium *Shewanella* sp. IRI-160 on non-targeted marine organisms, including adult and larval stages of copepods and oysters, as well as blue crab larvae. The results demonstrated smaller and earlier growth stages of these animals may be more sensitive to the algicide compared to those that have a larger size or are at later life stages. However, there were minimal or no effects on these species and life stages when evaluated at concentrations required to control the growth of dinoflagellates. Data resolving the impacts of algicidal bacteria or their products on non-target species and the ecosystem in general is essential to evaluating risk vs. benefits of their application and would be a necessary step toward their application for the control of HABs.

In addition to ecosystem impacts of algicidal bacteria, an important avenue for future research includes the isolation and characterization of individual algicidal compounds and confirmation of their activities. Using purified algicidal compounds over live bacteria may provide certain advantages, such as increased stability, as well as increasing cost-effectiveness in cases where the purified bacterial algicides are commercially available. In line with this effort, several compounds were isolated from bacteria that show algicidal activity. Some examples include amines, such as Nω- acetylhistamine from *Bacillus* sp. B1 (Zhu et al., 2021) and 2-isobutoxyphenylamine from a marine actinomycete (An et al., 2015), pigments prodigiosin from *Hahella* spp., or deinoxanthin from *Deinococcus* sp. which target a variety of HAB species (Nakashima et al., 2006; Li et al., 2015; Zhang et al., 2017, 2020b), as well as mycosubtilins from *Bacillus* sp. (Jeong and Son, 2021). Identification of algicidal compounds may be facilitated by paired analysis of transcriptome, genome, and/or metabolome data from algicidal bacteria and/or their target algal species (e.g., Zhang et al., 2021a,b). In addition, most studies so far have evaluated the effects of individual compounds isolated from algicidal bacteria on algal species. However, even in the case where multiple compounds were purified (e.g., Umetsu et al., 2019; Jeong and Son, 2021; Rose et al., 2021), only a few studies evaluated the combined effects of bacterial algicidal compounds (e.g., Guo et al., 2015; Zhuang et al., 2018; Ternon et al., 2019; Wang, 2021). These studies demonstrated that algicidal compounds, when applied in combination, may yield a higher activity compared to each of the individual components, illustrating the potential complexity of bacterial algicides and the importance of evaluating these compounds in combination.

Additional research should also be directed to evaluate the capability of algicidal bacteria and their algicides to control HABs under environmentally relevant conditions. In line with this, studies that focus on the development of modeling tools to better understand the algae-bacteria interactions may provide a bridge for laboratory experiments to be conducted under more environmentally relevant conditions. A good example is the use of microfluidic platforms that integrate microbial interactions and multiple environmental factors, and which can be coupled with mathematical modeling to understand the microbial community dynamics in a quantitative manner (reviewed by Liu et al., 2021).

Finally, development of application technologies requires studies of their efficacy in environments at risk for HABs. These evaluations are necessary not only to assess the activity of algicidal bacteria but also to identify any unexpected effects that may increase risk to the environment. The ability to conduct these field experiments, however, often relies on the approval of government agencies, where permitting requirements may differ based on location. For instance, clays have been dispersed in the field to flocculate HABs in many countries including Japan, South Korea, and Australia for over two decades (reviewed by Park et al., 2013), and have also been employed in China as a common method to control HABs since 2014 (reviewed by Yu et al., 2017). However, this approach has not been widely adopted in other countries, due in large part to concerns associated with their negative effects on the ecosystem (Brown et al., 2019). With the emerging impacts and increased awareness of HAB events (Hallegraeff et al., 2021), additional research to critically evaluate the risks vs. benefits of algicide application may alleviate some concerns. This is particularly the case for the areas that have been impaired by HABs, where the benefits of using bacterial algicides may exceed any risks.

## Author Contributions

KC conceived the manuscript. All authors contributed to writing the manuscript.

## Conflict of Interest

The authors declare that the research was conducted in the absence of any commercial or financial relationships that could be construed as a potential conflict of interest.

## Publisher’s Note

All claims expressed in this article are solely those of the authors and do not necessarily represent those of their affiliated organizations, or those of the publisher, the editors and the reviewers. Any product that may be evaluated in this article, or claim that may be made by its manufacturer, is not guaranteed or endorsed by the publisher.

## Supplementary Material

Supplementary Table 1 Detailed list of papers reviewed, 2016-2021.
